# Electronic equivalent of a mechanical impact oscillator

**DOI:** 10.1038/s41598-025-23489-8

**Published:** 2025-11-13

**Authors:** Volodymyr Denysenko, Marek Balcerzak, Artur Dabrowski

**Affiliations:** Division of Dynamics, Lodz University of Technology, Lodz, 90-924 Poland

**Keywords:** Impact oscillator, Discontinuous system, Electronic equivalent circuit, Engineering, Mathematics and computing, Physics

## Abstract

This study proposes a novel electronic circuit design that accurately replicates the dynamics of a mechanical discontinuous impact oscillator characterized by hard elastic impacts. The governing equations of the circuit are systematically derived to establish its equivalence with the mechanical system. Numerical simulations are conducted for both single and coupled configurations, demonstrating a high degree of agreement between the electronic and mechanical models.

**Supplementary Information:**

The online version contains supplementary material available at 10.1038/s41598-025-23489-8.

## Introduction

The piecewise-linear impact oscillator^1^ is one of the simplest discontinuous mechanical systems. While the dynamics of a continuous linear oscillator are straightforward and predictable, the introduction of impacts enables the appearance of a wide range of complex behaviors, including period doubling and even chaotic solutions. Several practical applications of such systems have been reported. For instance, impact oscillator-inspired constructions have been employed as drive systems^2^. The drive described in^2^ consists of a capsule containing an internal linear oscillator attached to one wall of the capsule, capable of colliding with the opposite wall. By appropriately selecting the system parameters, controlled capsule motion can be achieved. Another potential application of modified impact oscillator systems is energy harvesting^3^. For example, a triboelectric energy harvester based on a three-degree-of-freedom vibro-impact oscillator has been reported in^3^. A further modification, referred to as a springless vibration energy harvester, was presented in^4^. Additionally, an energy harvester based on a nonlinear impact oscillator system, known as the electromagnetic cantilever-based broad-bandwidth frequency energy harvester, was introduced in^5^. Moreover, impact oscillators—particularly those exhibiting chaotic solutions—offer valuable insights for studying chaotic synchronization in discontinuous systems^6^.

Although experiments with real mechanical oscillators provide the most direct means of investigating their dynamics, electronic analogues are often preferred^7–9^. This approach offers several notable advantages, especially in the context of synchronization studies. First, stated research typically involves identical or nearly identical oscillators, which are much more easily constructed using electronic circuits than mechanical components^10^. Second, the implementation of different coupling schemes—particularly asymmetric or master–slave configurations—is significantly more challenging in mechanical setups, whereas electronic systems allow for precise and flexible realization of these schemes^11^. Another advantage is the ease with which system parameters can be adjusted in electronic circuits, using variable capacitors or resistors^12^. Finally, for impact oscillators, electronic implementations allow the realization of perfectly elastic collisions—an idealization that is extremely difficult to achieve in mechanical systems.

Different approaches to electronic impact oscillator circuit design can be found in the literature. The electric impact oscillator described in^13^ models soft collisions – by employing a spring instead of a rigid wall. Another electronic impact oscillator was proposed in^14^; however, despite exhibiting similar dynamical behaviour, its dynamics cannot be described by the same equations as those governing the mechanical impact oscillator. Therefore, it cannot be considered a true analogue of the mechanical system.

In this article, a novel electronic circuit, that is equivalent to a mechanical oscillator with perfectly elastic hard collisions - i.e., one in which the sign of the oscillator’s velocity is instantly reversed at impact^15^, is proposed. The key advantage of the presented design is that its dynamic behavior closely replicates that of the mechanical system. This makes it a practical alternative for experimental studies, particularly in investigations involving networks of coupled identical impact oscillators, where it greatly simplifies the experimental setup.

To demonstrate the equivalence between the mechanical impact oscillator model and the proposed circuit, we derive the governing equations for the electronic oscillator. Next, we present numerical simulations for the developed electronic circuit, in both single and coupled configurations. Additionally, the results of the circuit simulation, which replicates the single impact oscillator, are compared with the analytical solution of the corresponding mathematical model of mechanical oscillator. The results demonstrate strong correspondence between the circuit and the original mechanical system.

## Mechanical impact oscillator

**Fig. 1:**
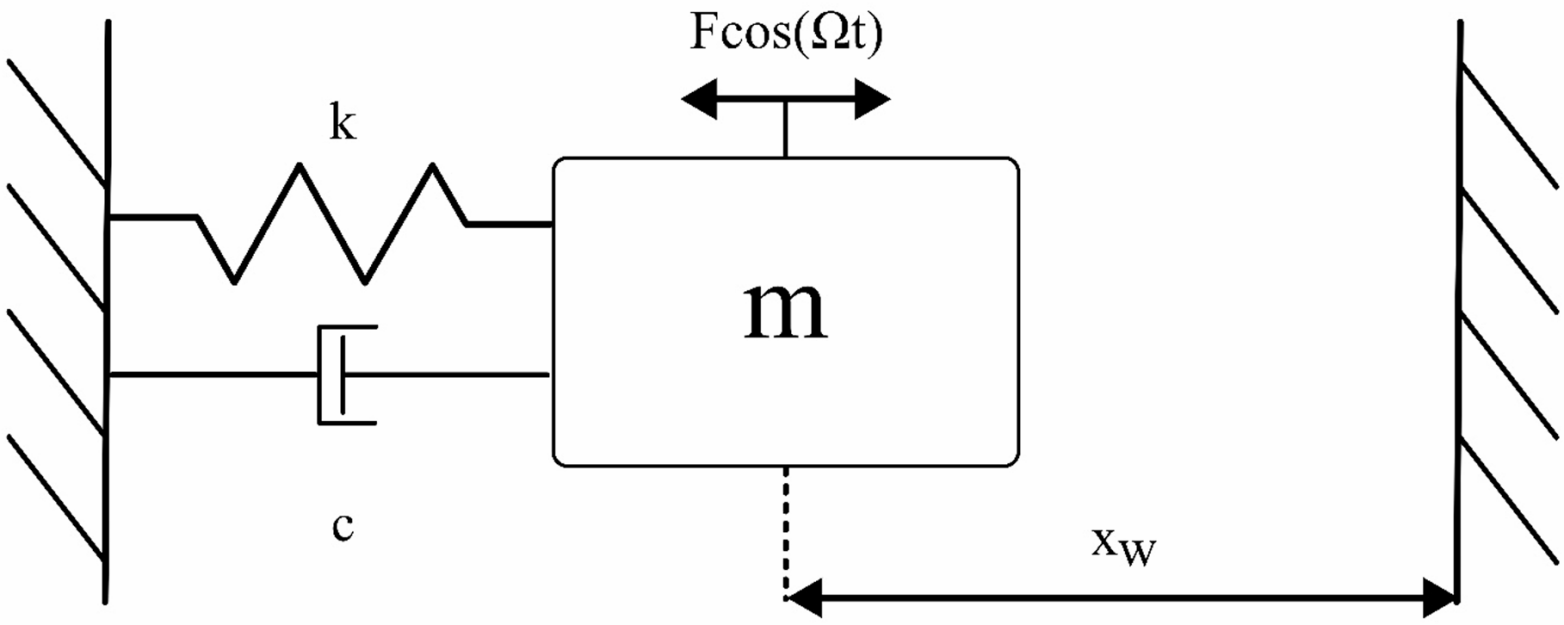
Impact mechanical oscillator.

The mechanical impact oscillator (Fig. 1), which is the typical mass-spring-damper system, capable of impacting a wall placed at the position $\:{X}_{w}$, is described by the following 2nd order differential equation:

1a $$\:m\ddot{X}+c\dot{X}+kX=F\:\text{c}\text{o}\text{s}\left({\Omega\:}t\right)$$

1b $$\:\begin{array}{c}\dot{X}\left({t}_{c}^{+}\right)=\:-R*\dot{X}\left({t}_{c}^{-}\right)\end{array},\:X\left({t}_{c}\right)={X}_{w}$$

where $\:X$ is the displacement of the oscillator, $\:m$ is its mass, $\:c$ is the damping coefficient, $\:k$ is the spring stiffness, and $\:F$ and Ω are the amplitude and frequency of the external forcing, respectively. $\:R$ is the coefficient of restitution, and $\:{t}_{c}$ is the moment in time when the collision with the wall occurs.

After reducing the Eq. (1) to the dimensionless form, the equations of motion are as follows:

2a $$\:{x}^{{\prime\:}{\prime\:}}\left(\tau\:\right)+2\zeta\:{x}^{{\prime\:}}\left(\tau\:\right)+x\left(\tau\:\right)=f\text{cos}\left(\eta\:\tau\:\right)$$

2b $$\:\begin{array}{c}{x}^{{\prime\:}}\left({\tau\:}_{c}^{+}\right)=\:-R*{x}^{{\prime\:}}\left({\tau\:}_{c}^{-}\right)\end{array},\:x\left({\tau\:}_{c}\right)={x}_{w}$$

where $\:\zeta\:=\frac{c}{2\sqrt{mk}}$ is the damping ratio, $\:\omega\:=\sqrt{\frac{k}{m}}$ is the natural frequency of the oscillator, $\:f=\frac{F}{m{\omega\:}^{2}}$, $\:\tau\:=\omega\:t$ is the dimensionless time, $\:\eta\:=\frac{{\Omega\:}}{\omega\:}$ is the dimensionless excitation frequency, $\:x=\frac{kX}{F}$ is the dimensionless displacement of the oscillator, $\:{x}_{w}=\frac{k{X}_{W}}{F}$ is the dimensionless position of the wall, and $\:{\tau\:}_{c}$ is the dimensionless time of collision.

### Equivalent circuit

**Fig. 2:**
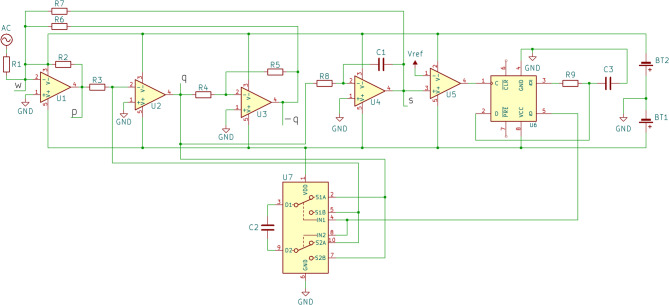
The proposed electronic circuit, equivalent to Eq. (2).

The diagram of the designed circuit, equivalent to the mechanical impact oscillator described by Eq. (2), is presented in Fig. 2. The circuit consists of two parts. The first part, comprising operational amplifiers (U1–U4) configured as inverting amplifiers or inverting integrators^16^, represents a smooth linear oscillator without impacts. In the absence of the remaining components, its behavior would correspond to that of a collision-free oscillator. The second part, which includes a comparator (U5), a D-type flip-flop (U6), and an analog switch (U7), is responsible for impact detection and response. The comparator U5 detects collisions, the flip-flop U6 stores the collision state, and the switch U7 reverses the velocity sign at the moment of impact. The high-resolution version of the diagram shown in Fig. 2 can be found in Supplementary Material S1.

To demonstrate that the dynamics of the electronic circuit reproduce those described by Eq. (2), the circuit model must be derived in the form of a differential equation. This requires analyzing the voltages at points **p**, **q**, and **s**. The voltage at point **p**, which is the output of operational amplifier U1 configured as a summing amplifier^16^, is given by:

3 $$\:\begin{array}{c}{V}_{p}=-{R}_{2}\left(\frac{{V}_{AC}}{{R}_{1}}-\frac{{V}_{q}}{{R}_{6}}+\frac{{V}_{s}}{{R}_{7}}\right)\end{array}$$

where $\:{V}_{AC}$ is the voltage of an external source. Operational amplifiers U2 and U4 are configured as integrators; therefore, the rate of change of the voltage at points **q** and **s**, which are the outputs of U2 and U4 respectively, can be described as follows.

4a $$\:\begin{array}{c}{\dot{V}}_{q}=-\frac{1}{{R}_{3}{C}_{2}}{V}_{p}\end{array}$$

4b $$\:\begin{array}{c}{\dot{V}}_{s}=-\frac{1}{{R}_{8}{C}_{1}}{V}_{q}\end{array}$$

Substituting Eq. (3) into Eq. (4a) yields the following system of differential Eq. 

5a $$\:\begin{array}{c}{\dot{V}}_{s}=-\frac{1}{{R}_{8}{C}_{1}}{V}_{q}\end{array}$$

5b $$\:\begin{array}{c}{\dot{V}}_{q}=-\frac{{R}_{2}}{{R}_{3}{C}_{2}}\left(\frac{{V}_{AC}}{{R}_{1}}-\frac{{V}_{q}}{{R}_{6}}+\frac{{V}_{s}}{{R}_{7}}\right)\end{array}$$

By differentiating Eq. (5a), the following expression is derived.

6 $$\:\begin{array}{c}{-R}_{8}{C}_{1}{\ddot{V}}_{s}={\dot{V}}_{q}\end{array}$$

Inserting Eq. (5a) and Eq. (6) into Eq. (5b) results in the following differential equation of the 2nd order.

7 $$\:\begin{array}{c}{\ddot{V}}_{s}=\frac{{R}_{2}}{{R}_{8}{C}_{1}{R}_{3}{C}_{2}}\left(\frac{{V}_{AC}}{{R}_{1}}+\frac{{R}_{8}{C}_{1}{\dot{V}}_{s}}{{R}_{6}}+\frac{{V}_{s}}{{R}_{7}}\right)\end{array}$$

Assuming $\:{V}_{s}=-x,\:{R}_{3}{C}_{2}={R}_{8}{C}_{1}={\omega\:}^{-1}=1,\:\frac{{R}_{2}}{2{R}_{6}}=\zeta\:,\:{R}_{7}={R}_{2}={R}_{1},\:{V}_{AC}={V}_{A}\text{c}\text{o}\text{s}\left({\Omega\:}\text{t}\right)$, Eq. (7) can be rewritten in the following form.

8 $$\:\begin{array}{c}\ddot{x}+2\zeta\:\omega\:\dot{x}+{\omega\:}^{2}x\:={V}_{A}{{\upomega\:}}^{2}\text{cos}\left({\Omega\:}\text{t}\right)\end{array}$$

Next, introducing non-dimensional time $\:\tau\:=\omega\:t$, Eq. (8) can be simplified as follows:

9 $$\:\begin{array}{c}{x}^{{\prime\:}{\prime\:}}\left(\tau\:\right)+2\zeta\:{x}^{{\prime\:}}\left(\tau\:\right)+x\left(\tau\:\right)={V}_{A}\text{cos}\left(\eta\:\tau\:\right)\end{array}$$

where $\:\eta\:=\frac{{\Omega\:}}{\omega\:}$ is non-dimensional frequency of the external excitation. As can be seen, Eq. (9) is identical to Eq. (2a), confirming that the proposed circuit accurately replicates the dynamics of a mechanical linear oscillator. Since $\:{V}_{s}=-x$, the voltage $\:{V}_{s}$ at point **s** corresponds to the negative position of the impact oscillator. From Eq. (4b) or (5a), one can observe that $\:{V}_{q}$ at point **q** represents the oscillator’s velocity. Finally, Eq. (4a) indicates that the voltage $\:{V}_{p}$ at point **p** corresponds to the negative acceleration of the oscillator.

Next, the impact with the wall, described by the Eq. (2b), must be implemented in the circuit. To detect the equivalent of a collision with the wall, a voltage comparator U5 was applied to compare $\:{V}_{s}$, representing the negative position of the oscillator, with the reference voltage, applied to the inverting input $\:{V}_{-}$ of the U5 comparator, which corresponds to the position of the wall $\:{x}_{w}.$ This, in turn, toggles the states of the analog switch U7, which is connected to capacitor C2. Since C2 forms part of the integrator circuit based on operational amplifier U2, the switching action effectively reverses the connections of the non-polarized capacitor C2. As a result, the sign of the voltage $\:{V}_{q}$​, which corresponds to the velocity of the mechanical oscillator, is inverted.

Consequently, the proposed circuit successfully replicates the behavior of a mechanical impact oscillator with a coefficient of restitution $\:R=1$.

## Numerical verification

To verify the equivalence between the proposed circuit design and the mechanical system described by Eq. (2), the system trajectories obtained from the circuit were compared with those derived from analytical solutions of Eq. (2). To ensure that the verification is both general and robust, system parameters were selected to represent cases exhibiting both chaotic and periodic behavior. The parameters of the system were taken from article^17^, which presents the Largest Lyapunov exponent (LLE) across a range of parameters and includes a bifurcation diagram for the linear impact oscillator.

The selected parameter values are: $\:\beta\:=0.05,\:{x}_{w}=2.0,\:R=1.0$, while $\:\eta\:$ is considered as a bifurcation parameter. Three values of $\:\eta\:$ were chosen to represent distinct dynamical regimes: $\:\eta\:=0.70$, which corresponds to the periodic solution without wall impact, $\:\eta\:=0.712$, which corresponds to the chaotic solution, and $\:\eta\:=0.74$, which corresponds to the period-3 solution.

To evaluate the suitability of the proposed circuit for investigating complete synchronization stability, we utilize a property of diagonally coupled systems described in^18^. A generalized system of two diagonally coupled arbitrary oscillators can be expressed as:

10a $$\:\begin{array}{c}\dot{\varvec{x}}=f\left(\varvec{x}\right)+{k}_{1}D\left(\varvec{y}-\varvec{x}\right)\end{array}$$

10b $$\:\begin{array}{c}\dot{\varvec{y}}=f\left(\varvec{y}\right)+{k}_{2}D\left(\varvec{x}-\varvec{y}\right)\end{array}$$

where $\:\varvec{x}\in\:{\mathbb{R}}^{n}$ and $\:\varvec{y}\in\:{\mathbb{R}}^{n}$ represent the state vectors of the first and second systems, respectively, with $\:n$ denoting the system order; $\:\varvec{f}:{\mathbb{R}}^{n}\to\:{\mathbb{R}}^{n}$ is the vector field describing the system dynamics; $\:\varvec{D}$ is a diagonal coupling matrix; and $\:{k}_{1}$, $\:{k}_{2}$ are the coupling coefficients. It has been shown in^18^ that the systems tend toward complete synchronization if the sum of the coupling coefficients exceeds the LLE of the uncoupled system, i.e., $\:{k}_{1}+{k}_{2}>{\lambda\:}_{LLE}$. Thus, the synchronization behavior—whether coherent or incoherent—depends on the LLE of the individual oscillator. This property can be used to validate the behavior of a system composed of two coupled electronic oscillators. To simplify the design of the two-oscillator circuit while preserving the described feature, unidirectional coupling is proposed $\:{(k}_{1}=0,\:{k}_{2}=k)$. Corresponding system of dimensionless differential equations is given below.

11a $$\:\begin{array}{c}{x}_{1}^{{\prime\:}}\left(\tau\:\right)={x}_{2}\left(\tau\:\right)\end{array}$$

11b $$\:\begin{array}{c}{x}_{2}^{{\prime\:}}\left(\tau\:\right)=-2\zeta\:{x}_{2}\left(\tau\:\right)-{x}_{1}\left(\tau\:\right)+f\text{cos}\left(\eta\:\tau\:\right)\end{array}$$

11c $$\:\begin{array}{c}\begin{array}{c}{x}_{2}\left({\tau\:}_{c}^{+}\right)=\:-R{x}_{2}\left({\tau\:}_{c}^{-}\right)\end{array},{x}_{1}\left({\tau\:}_{c}\right)={x}_{w}\end{array}$$

12a $$\:\begin{array}{c}{y}_{1}^{{\prime\:}}\left(\tau\:\right)={y}_{2}\left(\tau\:\right)+k\left({x}_{1}\left(\tau\:\right)-{y}_{1}\left(\tau\:\right)\right)\end{array}$$

12b $$\:\begin{array}{c}{y}_{2}^{{\prime\:}}\left(\tau\:\right)=-2\zeta\:{y}_{2}\left(\tau\:\right)-{y}_{1}\left(\tau\:\right)+f\text{cos}\left(\eta\:\tau\:\right)+k\left({x}_{2}\left(\tau\:\right)-{y}_{2}\left(\tau\:\right)\right)\end{array}$$

12c $$\:\begin{array}{c}\begin{array}{c}{y}_{2}\left({\tau\:}_{c}^{+}\right)=\:-R{y}_{2}\left({\tau\:}_{c}^{-}\right)\end{array},\:{y}_{1}\left({\tau\:}_{c}\right)={x}_{w}\end{array}$$

In order to simplify the design of the electronic circuit, equivalent to the Eqs. (12–13), this system can be reduced to the following pair of the second-order differential equations:

13a $$\:\begin{array}{c}{x}^{{\prime\:}{\prime\:}}\left(\tau\:\right)+2\zeta\:{x}^{{\prime\:}}\left(\tau\:\right)+x\left(\tau\:\right)=f\text{cos}\left(\eta\:\tau\:\right)\end{array}$$

13b $$\:\begin{array}{c}{x}^{{\prime\:}}\begin{array}{c}\left({\tau\:}_{c}^{+}\right)=\:-R{x}^{{\prime\:}}\left({\tau\:}_{c}^{-}\right)\end{array},\:x\left({\tau\:}_{c}\right)={x}_{w}\end{array}$$

14a $$\:\begin{array}{c}{y}^{{\prime\:}{\prime\:}}\left(\tau\:\right)+2\zeta\:{y}^{{\prime\:}}\left(\tau\:\right)+y\left(\tau\:\right)+k(1+2\zeta\:)({y}^{{\prime\:}}\left(\tau\:\right)-{x}^{{\prime\:}}\left(\tau\:\right))+\:2k\left(y\left(\tau\:\right)-x\left(\tau\:\right)\right)=f\text{cos}\left(\eta\:\tau\:\right)\end{array}$$

14b $$\:\begin{array}{c}{y}^{{\prime\:}}\begin{array}{c}\left({\tau\:}_{c}^{+}\right)=\:-R{y}^{{\prime\:}}\left({\tau\:}_{c}^{-}\right)\end{array},\:y\left({\tau\:}_{c}\right)={x}_{w}\end{array}$$

**Fig. 3:**
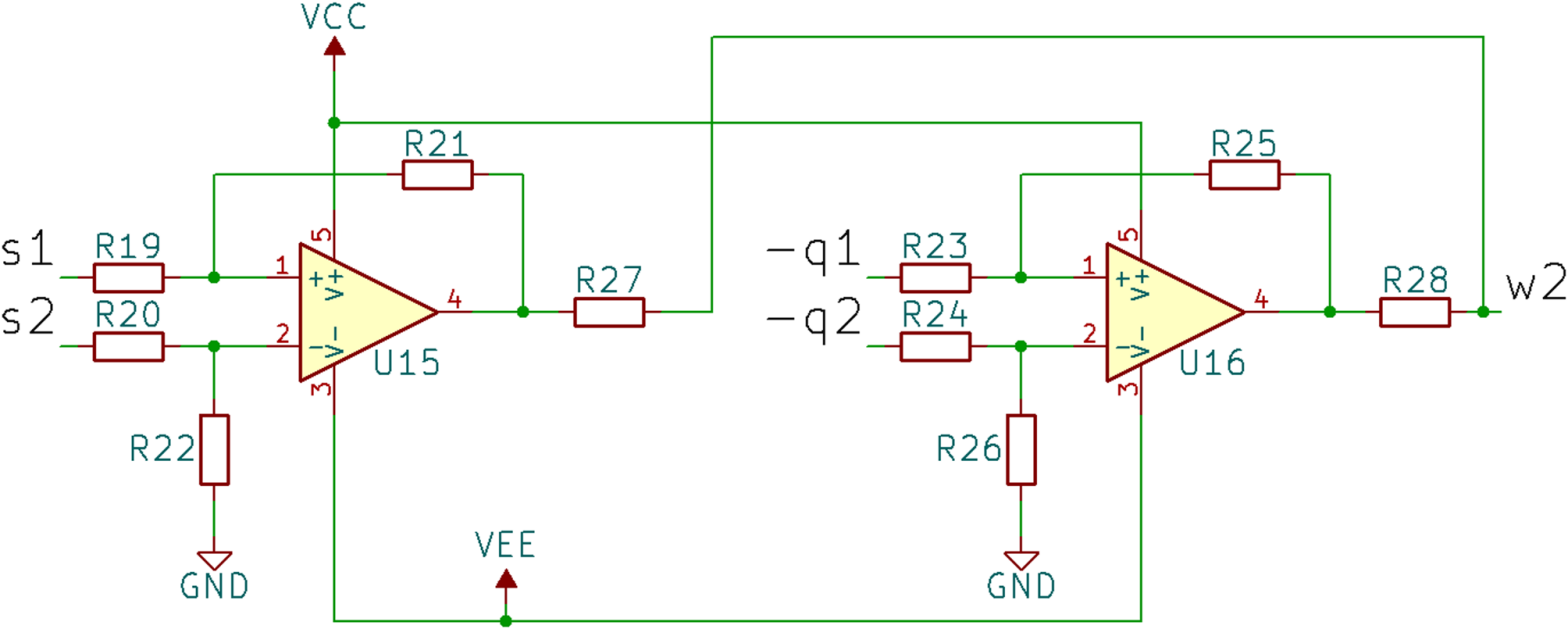
Diagram of the electronic circuit implementing unidirectional diagonal coupling from Eqs. (13–14).

Values of the system parameters in case of 2 coupled oscillators are the same as in case of the single oscillator with $\:\eta\:=0.712$. The electronic circuit equivalent to the system described by Eqs. (13)–(14) consists of two impact oscillator circuits connected via the coupling circuit, shown in Fig. 3. For the complete circuit diagram corresponding to the system described by Eqs. (13)–(14), see Supplementary Figure S2.

The coupling circuit is implemented using two operational amplifiers configured as differential amplifiers, whose outputs are proportional to the difference between their two inputs. As indicated in the previous section, the voltage signals at points **p**, **q** and **s** represent the negative acceleration, velocity, and negative displacement of the oscillator, respectively. For two coupled circuits, **s1** and **s2** correspond to the negative displacements in the first and second circuits, respectively, while **q1** and **q2** represent their velocities. Consequently, the output of the first differential amplifier (U15) corresponds to the difference between **s1** and **s2**, i.e., the position signals, resulting in the position coupling term $\:{y}_{1}-{x}_{1}$ (see Eq. (12a)). Similarly, the output of the second differential amplifier (U16) corresponds to the difference between **-q1** and **-q2**, i.e., the velocity signals, resulting in the velocity coupling term $\:{y}_{2}-{x}_{2}$ (see Eq. (12b)). Finally, the signal **w2** represents the sum of these coupling terms, which can be connected to the summing circuit, contributing to the left-hand side of Eq. (14a).

To simplify the circuit design, it is assumed that $\:{R}_{19}={R}_{20}={R}_{21}={R}_{22}$ and $\:{R}_{23}={R}_{24}={R}_{25}={R}_{26}$, ensuring that U15 and U16 yield unamplified differences in position and velocity, respectively. The outputs of U15 and U16 are then connected to the summing amplifier of the second oscillator via resistors $\:{R}_{27}$ and $\:{R}_{28}$, respectively.

By applying the same methodology used for the single oscillator, the dynamical model of the electronic circuit, consisting of two oscillators and the coupling, can be derived. The terms corresponding to the dynamics of a single oscillator remain the same in the electronic circuit, as in the case of the circuit representing a single oscillator. The coupling terms, when implemented electronically, are represented as follows:

15 $$\:\begin{array}{c}\frac{{R}_{11}}{{R}_{27}}=2k;\:\frac{{R}_{11}}{{R}_{28}}=k\left(1+2\zeta\:\right)\end{array}$$

The proposed equivalent electronic circuits - comprising both the single oscillator and the coupled oscillator configurations - were simulated using LTspice software^19^. For the single oscillator, simulations were performed over a time span corresponding to 200 periods of external excitation. For the coupled oscillators, the simulation duration was extended to 500 periods to capture long-term synchronization behavior.

Trajectories of the circuits, corresponding to the single oscillators were compared to the analytical solutions of the mechanical system, which model is described by Eqs. (2a-2b). To detect collisions, trajectory of the oscillator was evaluated every $\:{\Delta\:}t={10}^{-3}$ of the external excitation period. If a condition $\:x\left(t+{\Delta\:}t\right)>{x}_{w}$ was met, the exact moment of impact was determined using the bisection method.

Both circuits, fully prepared and configured for numerical simulations in LTspice software, are available in the repository^20^. The values of the resistances and capacitances for both the single- and two-oscillator circuits are provided in Supplementary Table S3.

## Results

**Fig. 4:**
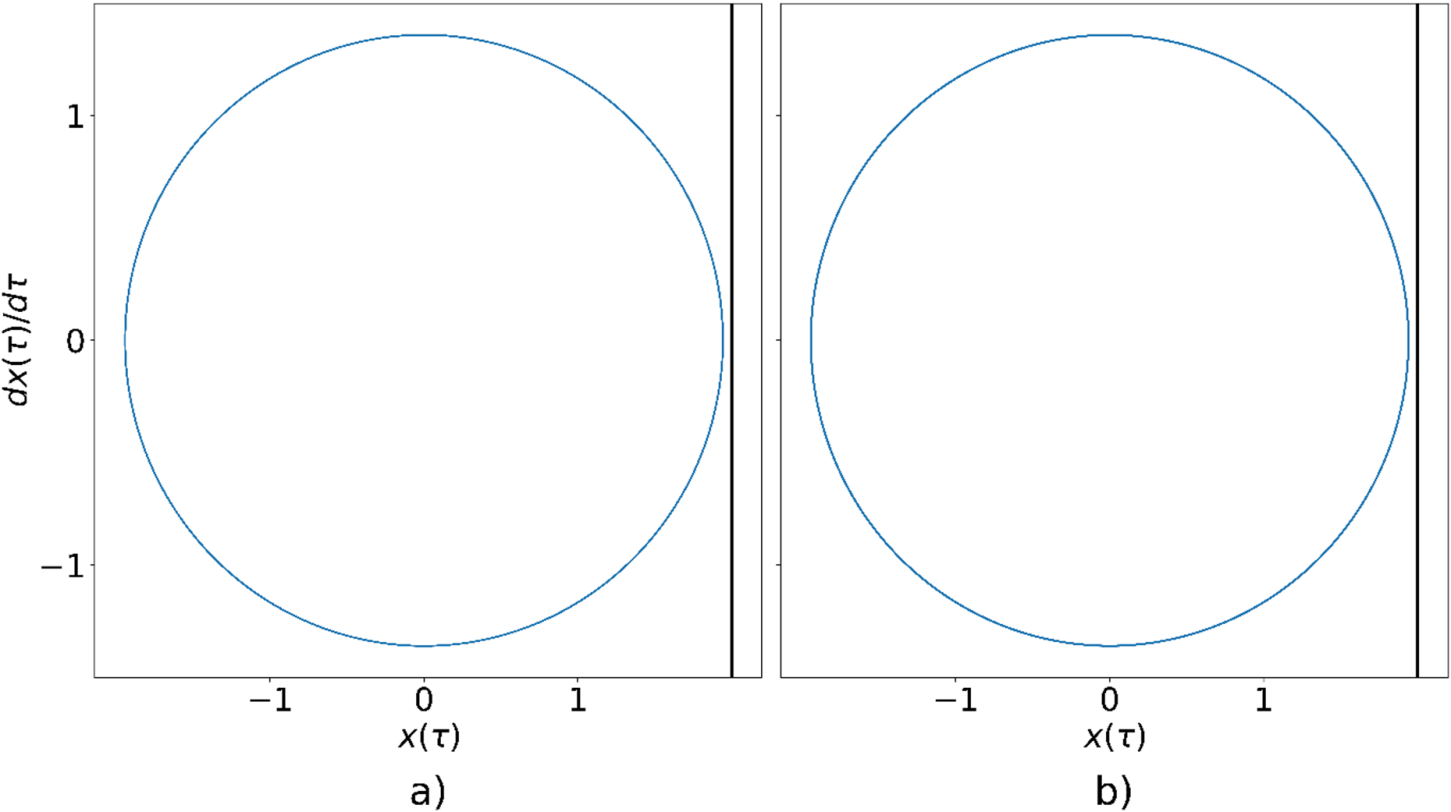
Phase-space trajectory of the oscillator for $\:{\upeta\:}=0.7$, obtained using: (**a**) numerical simulation, (**b**) circuit simulation.

The phase-space trajectory of the single oscillator for $\:\eta\:=0.7$, obtained analytically, is shown in Fig. 4a, while the corresponding trajectory from the circuit simulation is shown in Fig. 4b. It can be observed that, for this parameter value, the oscillator does not reach sufficient amplitude to collide the wall after the transient phase. The trajectories obtained by both methods are in excellent agreement, confirming the accuracy of the proposed circuit model.

**Fig. 5:**
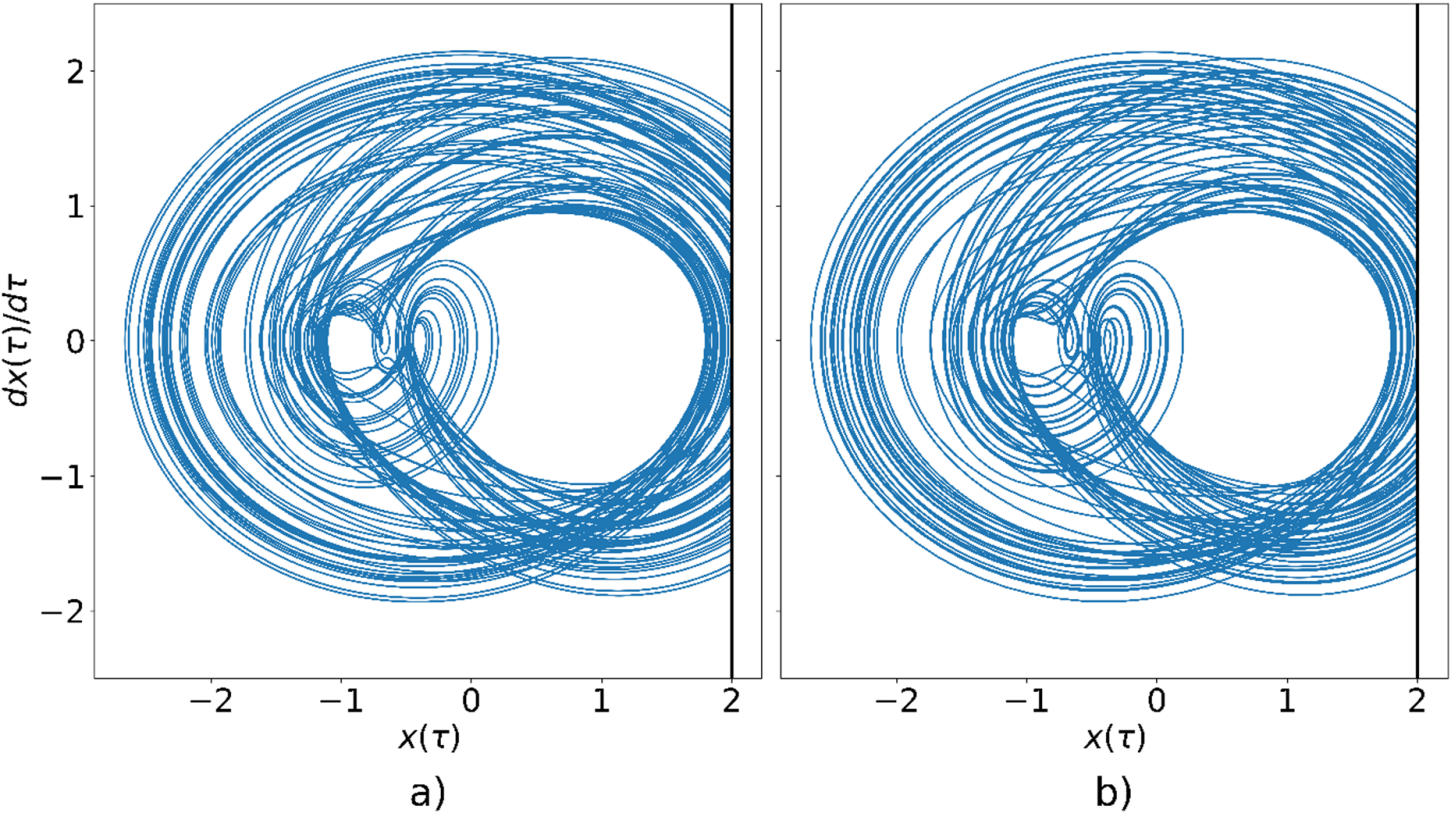
Phase-space trajectory of the oscillator for $\:{\upeta\:}=0.712$, obtained using: (**a**) numerical simulation, (**b**) circuit simulation.

Figure 5a and b present the phase-space trajectories of the impact oscillator for $\:\eta\:=0.712$, obtained via analytical solution and circuit simulation, respectively. Both methods yield a chaotic attractor, as expected for this parameter value. However, significant discrepancies between the attractors are evident. These differences are most likely attributable to numerical errors intrinsic to the circuit simulation, which are magnified by the pronounced sensitivity of chaotic systems to initial conditions and minute perturbations.

**Fig. 6:**
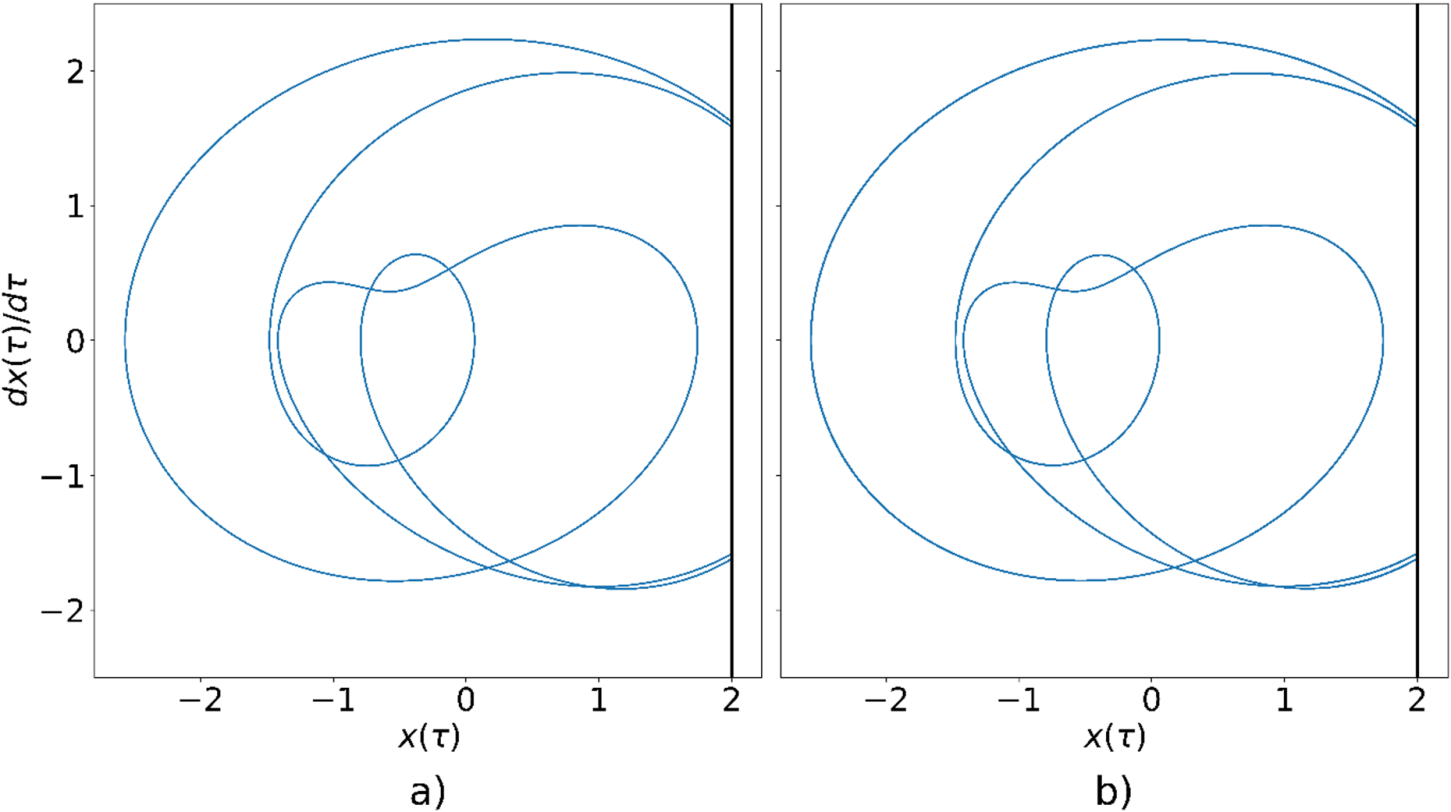
Phase-space trajectory of the oscillator for $\:{\upeta\:}=0.74$, obtained using: (**a**) numerical simulation, (**b**) circuit simulation.

Figure 6a displays the phase-space trajectory of the single oscillator for $\:\eta\:=0.74$, obtained via analytical solution, while Fig. 6b presents the corresponding trajectory derived from the circuit simulation. In both cases, the oscillator exhibits a period-3 solution. The resulting trajectories are nearly indistinguishable, thereby reinforcing the validity of the proposed electronic circuit in accurately reproducing the dynamics of the mechanical impact oscillator.

**Fig. 7:**
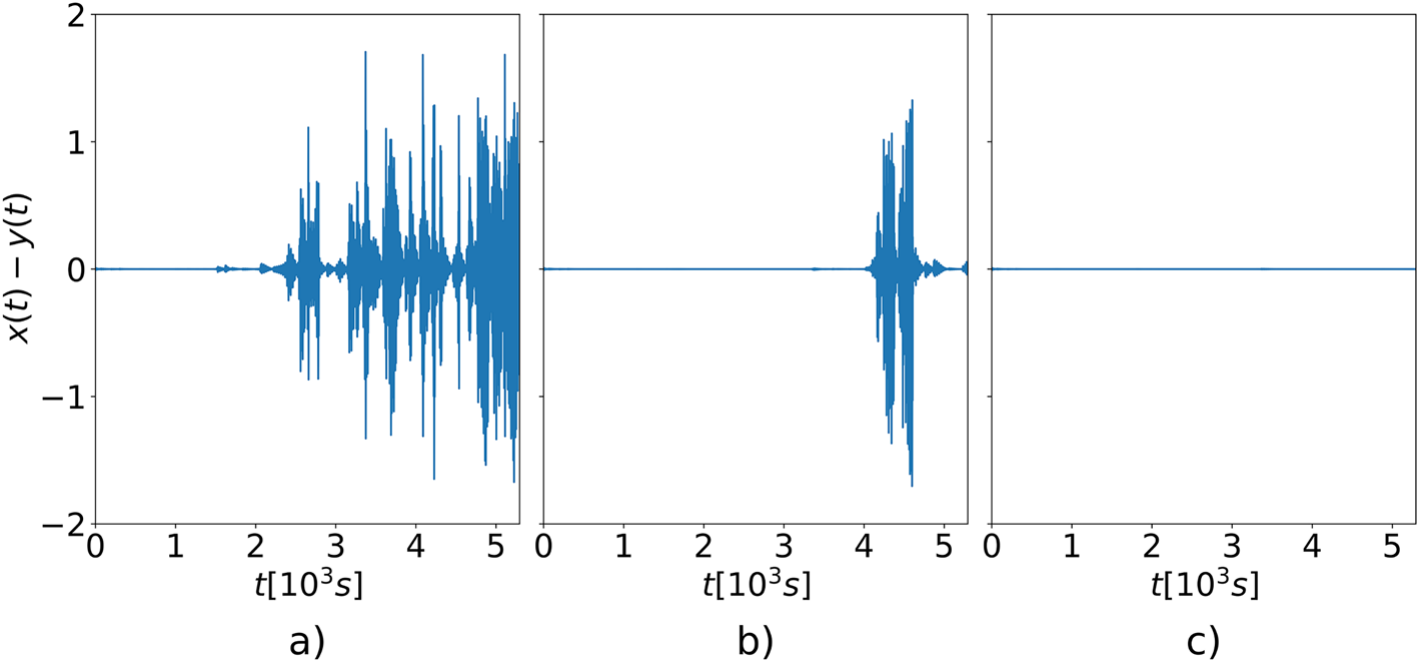
Time series of the position differences between two unidirectionally coupled oscillators for varying coupling coefficients: (**a**)$\:\:\text{k}=0.030$, (**b**) $\:\text{k}=0.031$, and (**c**) $\:\text{k}=0.032$.

Figure 7a, b and c illustrate the time series of the position differences between two unidirectionally coupled oscillators for coupling coefficients $\:k=0.030$, $\:k=0.031\:$and $\:k=\:0.032$, respectively. As shown, the oscillators fail to synchronize in the first two case, while stable synchronization is achieved in the third. This observation suggests that the LLE of the single oscillator lies between last two values, i.e., $\:0.031<{\lambda\:}_{LLE}<0.032$. The LLE value estimated using the numerical method described in^17^ is approximately equal to $\:{\lambda\:}_{LLE}\approx\:0.03$. Considering that the procedure used to estimate the LLE from the electronic oscillator is less precise than analytical methods, the result falls within an acceptable margin of error. An additional factor that may have contributed to the observed discrepancy is the numerical imprecision of the LTspice solver.

## Discussion

Based on the comparison between the trajectories obtained from the circuit simulation of the single oscillator and those derived from numerical simulations, it can be concluded that the trajectories are nearly indistinguishable in regimes exhibiting periodic behavior. Notable discrepancies emerge exclusively within the chaotic regime, which can be attributed to both the inherent sensitivity of chaotic systems and numerical inaccuracies introduced by the electronic circuit simulator. Nevertheless, the qualitative dynamics of the equivalent circuit remain consistent with those observed in numerical simulations.

Furthermore, the LLE of the equivalent circuit was estimated using a synchronization-based method. Although the obtained value slightly deviates from the LLE calculated via numerical and analytical approaches, the discrepancy remains within acceptable bounds. This is because the more accurate analytical estimation was applied to the numerical model of the impact oscillator, whereas the synchronization-based method was employed for the electronic implementation.

Overall, the results presented for both the single oscillator and the system of two diagonally coupled oscillators demonstrate that the proposed circuit faithfully reproduces the dynamics of the mechanical piecewise-linear impact oscillator. This confirms that the circuit design is functionally equivalent to its mechanical counterpart and can therefore be effectively employed in experimental investigations of both individual impact oscillators and networks of coupled impact oscillators. This approach offers two principal advantages: first, it enables high reproducibility in the realization of identical oscillators, which is essential for the experimental validation of coupled oscillator networks; second, it eliminates stochastic influences inherent to mechanical systems, such as friction and wear.

However, it is important to acknowledge that the proposed circuit design exhibits certain limitations. First, the range of signals—representing both displacement and velocity—is constrained by the supply voltage of the operational amplifiers employed. Second, the current design is not suitable for modeling the dynamics of impact oscillators involving inelastic or soft collisions. Nevertheless, based on both the system dynamics and the simulation results presented in Figs. 4, 5 and 6, the position and velocity of the oscillator remain bounded. This implies that the supply voltage may be adjusted if the initially selected value proves insufficient. It is also worth noting that the circuit was explicitly designed to replicate elastic soft collisions; therefore, the aforementioned limitation does not compromise the intended scope of the proposed experimental investigations.

Taken together, the proposed electronic circuit offers a robust and experimentally viable alternative to mechanical impact oscillators with elastic collisions, enabling precise and repeatable investigations of complex dynamical behaviors. This approach facilitates experimental exploration of coupled networks of discontinuous systems, while significantly simplifying the experimental setup and enhancing reproducibility.

## Supplementary Information

Below is the link to the electronic supplementary material.

Supplementary Material 1

Supplementary Material 2

Supplementary Material 3

## Data Availability

All circuit files prepared and configured for numerical simulations, using LTspice, along with the script developed in this study, are available in the research data linked to this paper^20^.
