# Supplementary figures and images for: Electronic equivalent of a mechanical impact oscillator
Source: Sci Rep. 2025 Nov 13;15:39853. doi: 10.1038/s41598-025-23489-8 (PMC12615831; doi:10.1038/s41598-025-23489-8)

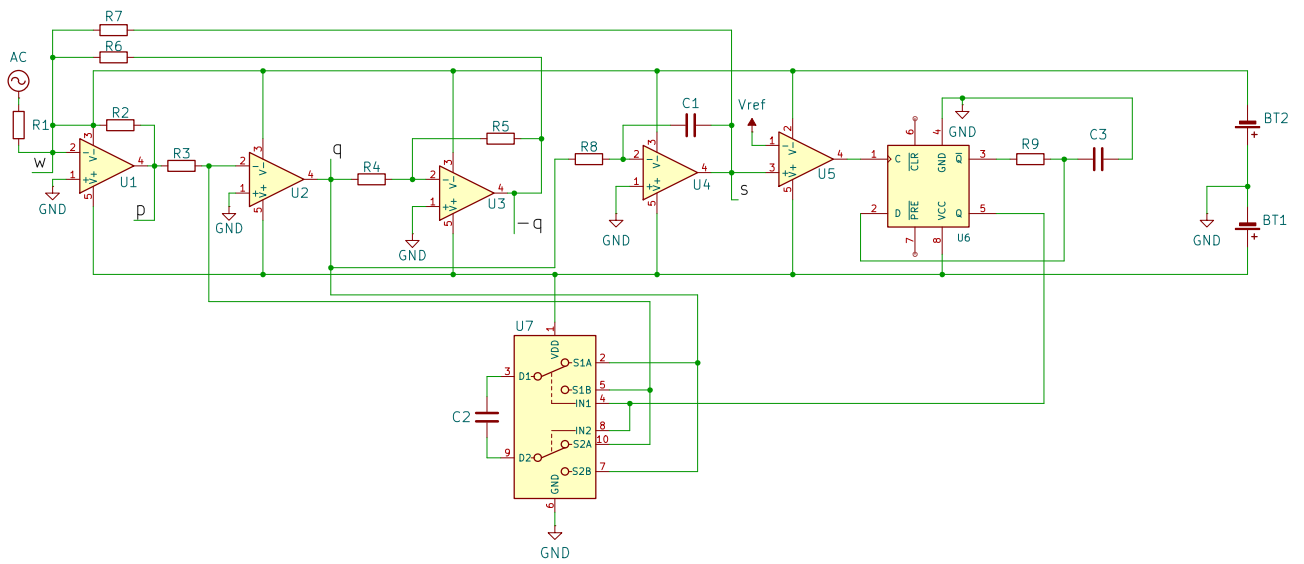

Supplement: Supplementary file 1 — Supplementary Material 1 [file 41598_2025_23489_MOESM1_ESM.pdf]

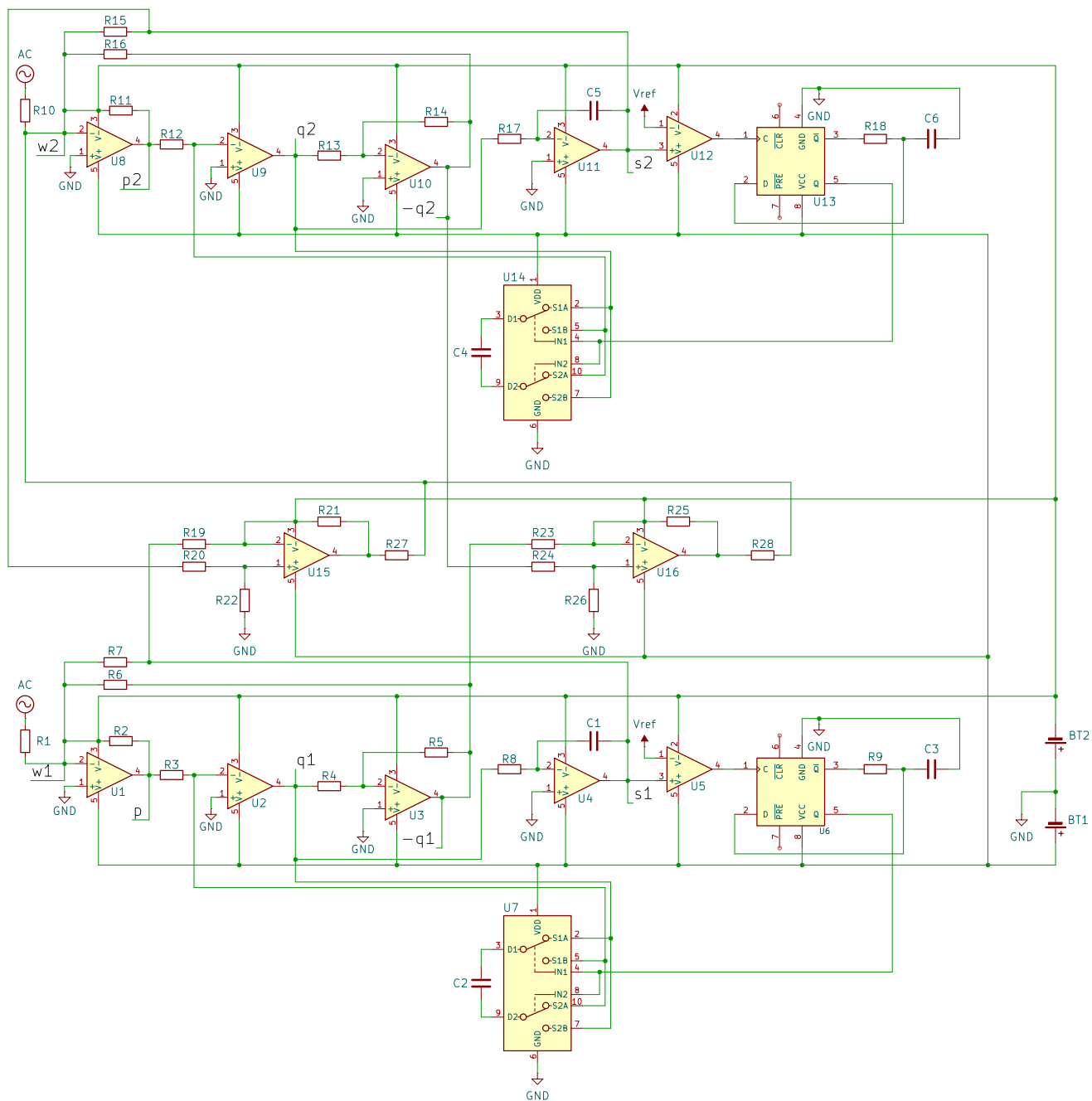

Supplement: Supplementary file 2 — Supplementary Material 2 [file 41598_2025_23489_MOESM2_ESM.pdf]
