# Supplementary material for: Electronic equivalent of a mechanical impact oscillator
Source: Sci Rep. 2025 Nov 13;15:39853. doi: 10.1038/s41598-025-23489-8 (PMC12615831; doi:10.1038/s41598-025-23489-8)
Supplement: Supplementary file 3 — Supplementary Material 3 [file 41598_2025_23489_MOESM3_ESM.pdf]

Table 1 Resistance and capacitance values in the single oscillator circuit.

R1	47k
R2	47k
R3	1000k
R4	47k
R5	47k
R6	470k
R7	47k
R8	1000k
R9	100
R10	47k
R11	47k
R12	1000k
R13	47k
R14	47k
R15	470k
R16	47k
R17	1000k
R18	100
R19	47k
R20	47k
R21	47k
R22	47k
R23	47k
R24	47k
R25	47k
R26	47k
R27 ($\sigma \approx 0.031$)	758k
R28 ($\sigma \approx 0.031$)	1378k
R27 ($\sigma \approx 0.032$)	735k
R28 ($\sigma \approx 0.032$)	1335k
C1	1µ
C2	1µ
C3	330n
C4	1µ
C5	1µ
C6	330n
